# Glutamine reliance in cell metabolism

**DOI:** 10.1038/s12276-020-00504-8

**Published:** 2020-09-17

**Authors:** Hee Chan Yoo, Ya Chun Yu, Yulseung Sung, Jung Min Han

**Affiliations:** 1 Yonsei Institute of Pharmaceutical Sciences, College of Pharmacy, Yonsei University, Incheon, 21983 South Korea; 2 Department of Integrated OMICS for Biomedical Science, Yonsei University, Seoul, 03722 South Korea

**Keywords:** Cancer metabolism, Metabolomics, Cancer metabolism, Metabolomics

## Abstract

As knowledge of cell metabolism has advanced, glutamine has been considered an important amino acid that supplies carbon and nitrogen to fuel biosynthesis. A recent study provided a new perspective on mitochondrial glutamine metabolism, offering mechanistic insights into metabolic adaptation during tumor hypoxia, the emergence of drug resistance, and glutaminolysis-induced metabolic reprogramming and presenting metabolic strategies to target glutamine metabolism in cancer cells. In this review, we introduce the various biosynthetic and bioenergetic roles of glutamine based on the compartmentalization of glutamine metabolism to explain why cells exhibit metabolic reliance on glutamine. Additionally, we examined whether glutamine derivatives contribute to epigenetic regulation associated with tumorigenesis. In addition, in discussing glutamine transporters, we propose a metabolic target for therapeutic intervention in cancer.

## Amino acid metabolism: Glutamine in healthy and cancerous cells

Insights into how the amino acid glutamine powers cellular metabolism could pave the way for effective therapeutic strategies for ‘starving’ tumor cells. Healthy cells can manufacture enough glutamine to sustain normal function, but cancerous growth creates heavier demand for this important molecule. Jung Min Han and colleagues at Yonsei University in Incheon, South Korea have reviewed the various cellular functions of glutamine, and discuss opportunities to cut off supply and thereby derail tumor proliferation. Glutamine serves as a building block both for amino acids and nucleic acids, and is also consumed during mitochondrial energy production. Several groups are exploring the feasibility of inactivating glutamine synthesis or halting cellular uptake of this amino acid as a means of depriving cancer cells of nutrients. A deeper understanding of glutamine’s metabolic functions should accelerate progress on this front.

## Introduction

After Otto Warburg discovered that cancer cells exhibit significantly elevated glucose consumption and lactate secretion even in the presence of oxygen^1^, studies on cell metabolism have accumulated. The major findings are that aerobic glycolysis is not a symptom of impaired mitochondrial function, and that glutamine supports mitochondrial oxidative metabolism when pyruvate derived from glucose is converted into lactate and secreted^2–4^. Glutamine, which is a nonessential amino acid (NEAA) due to the endogenous glutamine biosynthesis pathway, is currently considered essential in cancer cells because transformed cells consume glutamine at a rate exceeding that of glutamine biosynthesis^5^. Glutamine has a versatile role in cell metabolism, participating in tricarboxylic acid (TCA) cycle supplementation and the biosynthesis of nucleotides, glutathione (GSH), and other nonessential amino acids. Thus, glutamine deprivation suppresses cancer growth and even induces cell death in several cancers^6,7^. This metabolic dependency of transformed cells on glutamine constitutes the recently defined glutamine addiction^8^.

Since glutaminase 1 (GLS1), a key mitochondrial enzyme that catalyzes the deamidation of glutamine, was first discovered in the kidney in 1958^9^, many enzymes involved in glutamine metabolism have been reported^4^. In addition, glutamine has been confirmed to be a major nutrient source for oxidative metabolism in some cancer cell lines^10–12^, and specific genetic interference with glutaminase (GLS) inhibits tumor cell growth^13^. Moreover, CB-839, the first glutaminase inhibitor, has entered several clinical trials^14,15^. Despite the importance of mitochondrial glutamine metabolism, the mitochondrial glutamine transporter, encoded by a transcript variant of the SLC1A5 gene, which encodes a well-known plasma membrane glutamine transporter, was only recently discovered^16^. Thus, glutamine metabolism is intriguingly linked with intricate cell metabolic processes via enzymes associated with mitochondrial glutaminolysis, cytosolic glutamine metabolism, and glutamine-derived metabolites that perform diverse cellular functions.

In this review, we first introduce metabolic pathways that enable glutamine to respond to diverse cellular needs and then discuss the metabolic link by which glutamine-derived metabolites may affect cellular metabolic processes, including NEAA synthesis, epigenetic modifications, and hypoxia adaptation. We next discuss recent advances in glutamine metabolism with particular emphasis on tumorigenesis. We aim to offer both the principles underlying cellular dependence on glutamine metabolism under various conditions and a discussion of future directions that are leading our efforts to investigate the role of glutamine in cellular metabolism.

## Glutamine metabolic pathways

**Fig. 1:**
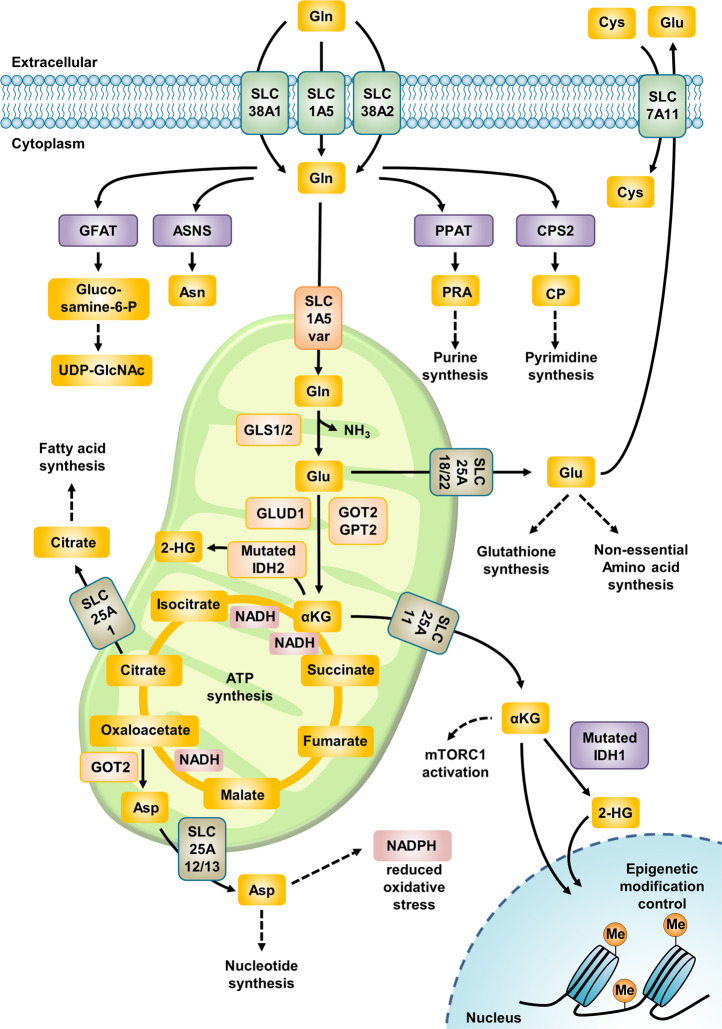
Glutamine metabolic pathways. Glutamine enters through several plasma membrane glutamine transporters and is then utilized in the cytosol in processes such as the biosynthesis of nucleotides, asparagine, and UDP-GlcNAc. For glutaminolysis, glutamine is transported into the mitochondrial matrix through the SLC1A5 variant and subsequently converted to glutamate by GLS. Next, GLUD1 or several aminotransferases catalyze the deamidation of glutamate, producing α-KG. Glutamine-derived α-KG supplies metabolites for the TCA cycle and fuels the generation of 2-HG under conditions of IDH2 mutation or hypoxia. Citrate derived from glutamine via reductive carboxylation supports fatty acid synthesis under conditions of hypoxia or HIF-2α transcription factor stabilization. Glutamine-derived α-KG also activates the mTORC1 pathway. Α-KG and 2-HG affect epigenetic modification through α-KG-dependent dioxygenases. Gln glutamine, Glu glutamate, Asn asparagine, Cys cystine, Asp aspartate, αKG α-ketoglutarate, PRA 5-phosphoribosyl-1-amine, CP carbamoyl phosphate, GFAT glutamine-fructose-6-phosphate transaminase, ASNS asparagine synthetase, PPAT phosphoribosyl pyrophosphate amidotransferase, CPS carbamoyl phosphate synthetase, GLS glutaminase, GLUD glutamate dehydrogenase, GOT glutamic-oxaloacetic transaminase, GPT glutamic-pyruvate transaminase, IDH isocitrate dehydrogenase, 2-HG 2-hydroxyglutarate, Me methylation.

Glutamine is transported into cells through plasma membrane glutamine transporters such as SLC1A5, SLC38A1, and SLC38A2^17^ and can then be used for the biosynthesis of hexosamine, nucleotides, and asparagine in the cytoplasm (Fig. 1). For mitochondrial glutaminolysis, cytosolic glutamine must be transported through the inner mitochondrial membrane via the SLC1A5 variant, a mitochondrial glutamine transporter^16^. Next, glutamine is converted into glutamate by GLSs, amidohydrolase enzymes that catalyze the conversion of glutamine into glutamate, releasing ammonium ions. GLSs have at least three isoforms, GLS1, GLS2, and GAC (a splicing isoform of GLS1), all of which were recently reported to be localized in mitochondria^18–20^. Mitochondrial glutamate generated via these catabolic pathways can be exported from mitochondria to the cytosol through the SLC25A18 and SLC25A22 transporters^21^, and cytosolic glutamate then participates in the biosynthesis of glutathione—a tripeptide comprising glutamate, cysteine, and glycine—and NEAAs (alanine, proline, aspartate, asparagine, and arginine) and is used as an exchange factor for importing extracellular cystine via SLC7A11. Mitochondrial glutamate is subsequently converted into alpha-ketoglutarate (α-KG) by glutamate dehydrogenase 1 (GLUD1 or GDH1) or by several mitochondrial aminotransferases, including glutamic-pyruvic transaminase 2 (GPT2) and glutamic-oxaloacetic transaminase 2 (GOT2). In addition, α-KG is exported from mitochondria through SLC25A11 to the cytosol^21^ and then participates in fatty acid biosynthesis and NADH generation^22^ (Fig. 1). Mitochondrial α-KG can then participate in the TCA cycle, supporting the oxidative phosphorylation (OXPHOS) pathway or the reductive carboxylation pathway^23^. In the oxidative phosphorylation pathway, metabolites of glutamine participate in the generation of an electron donor, such as NADH or FADH_2_, after synthesis of guanosine triphosphate (GTP) and adenosine triphosphate (ATP). In addition to pyruvate-derived acetyl-CoA, α-KG-derived metabolites (e.g., succinate and fumarate) generated via glutaminolysis are considered oncometabolites contributing to tumorigenesis^23^. Citrate, generated by reductive carboxylation of α-KG, is especially crucial for lipid synthesis under low-oxygen conditions^24,25^.

α-KG is considered an important cofactor for enzymes participating in epigenetic modification^26^. It is a substrate for α-ketoglutarate dehydrogenase (OGDH) in oxidative reactions generating succinyl-CoA and isocitrate dehydrogenase 1 (IDH1) or isocitrate dehydrogenase 2 (IDH2), which catalyze the reductive carboxylation reaction converting α-KG to isocitrate. Cancer cells in tumors with IDH1 or IDH2 mutations show oncogenic activity by converting glutamine-derived α-KG to 2-hydroxyglutarate (2-HG), which competitively inhibits α-KG-dependent histone and DNA modification enzymes^27^. Additionally, glutamine-derived aspartate plays a crucial role in hypoxic environments or environments with electron transport chain (ETC) impairment^28^. In addition, NADPH generation via glutamine metabolism in cancer cells supports redox homeostasis by maintaining the cytosolic NADPH pool used to restore oxidized glutathione^29^ (Fig. 1).

### Nucleotides synthesized from glutamine

**Fig. 2:**
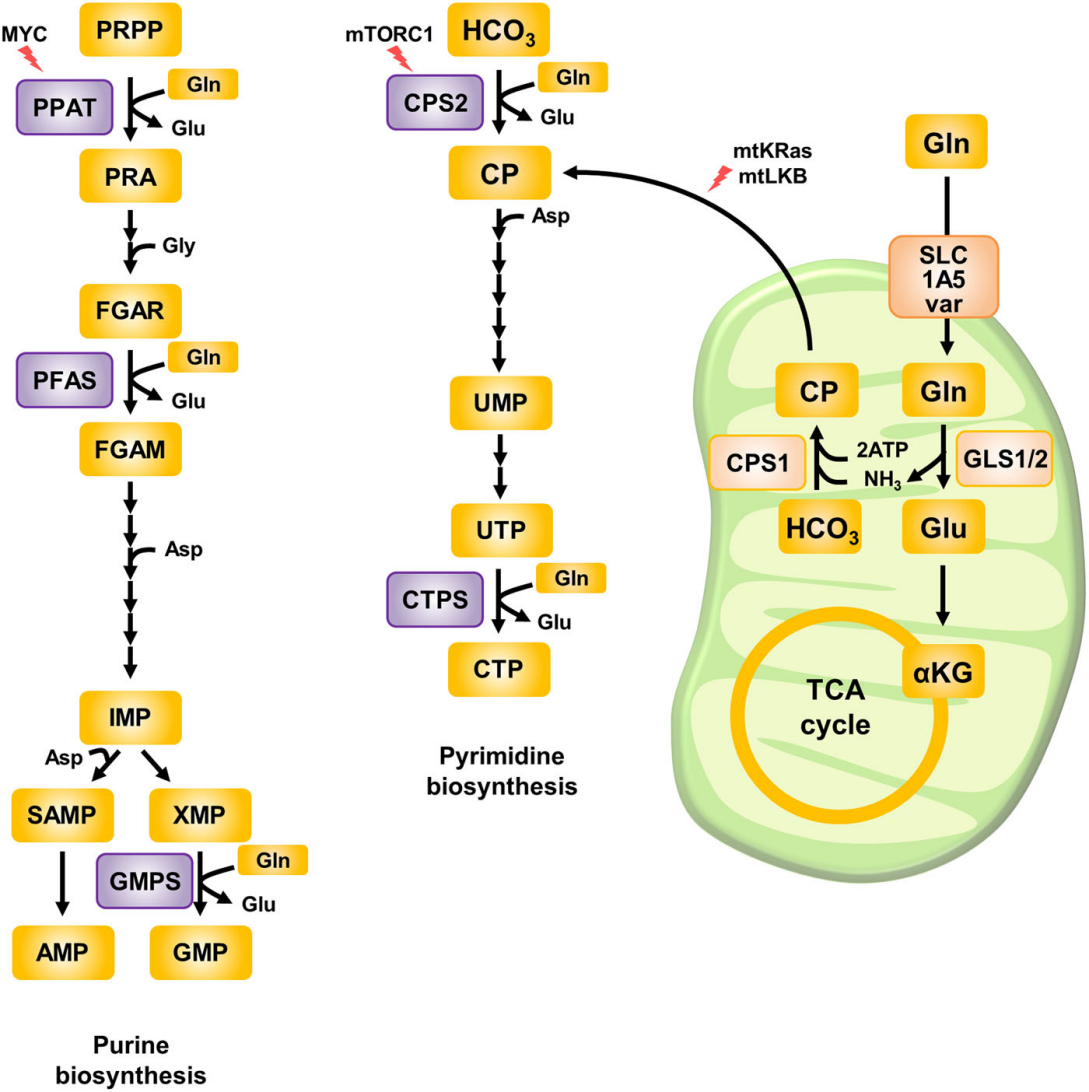
Nucleotide biosynthesis from glutamine. In purine biosynthesis, two glutamine molecules are consumed to synthesize AMP, and three glutamine molecules are used to synthesize GMP. Similarly, in pyrimidine biosynthesis, one glutamine molecule is consumed to synthesize UMP, and two glutamine molecules are spent to convert UTP into CTP. The initial step in de novo pyrimidine synthesis is the condensation reaction between glutamine and bicarbonate catalyzed by CPS to produce CP. In cells with an oncogenic mutational status, including K-Ras mutation, glutaminolysis sustains mitochondrial generation of CP by providing enough nitrogen fuel as ammonium ions, and mitochondrial CP then participates in cytosolic de novo pyrimidine synthesis. Glutamine-induced nucleotide biosynthesis is also enhanced by MYC or growth signals such as mTORC1 activation. PPAT phosphoribosyl pyrophosphate amidotransferase, PFAS phosphoribosylformylglycinamidine synthase, GMPS GMP synthetase, CPS carbamoyl phosphate synthetase, CTPS CTP synthetase, GLS glutaminase, PRPP 5-phosphoribosyl-1-pyrophosphate, PRA 5-phosphoribosyl-1-amine, FGAR N2-formyl-N1-(5-phospho-d-ribosyl)glycinamide, FGAM 2-(formamido)-N1-(5-phospho-d-ribosyl)acetamidine, IMP inosine monophosphate, SAMP adenylosuccinate, XMP xanthosine monophosphate, AMP adenosine monophosphate, GMP guanosine monophosphate, CP carbamoyl phosphate, UMP uridine monophosphate, UTP uridine triphosphate, CTP cytidine, Glu glutamine, Glu glutamate, αKG α-ketoglutarate.

Cytosolic glutamine supports nucleotide biosynthesis, which is essential for rapidly proliferating cells^30^. The gamma-nitrogen of glutamine is used in five reactions in de novo nucleotide synthesis, and its bioavailability controls de novo biosynthesis of pyrimidines and purines (Fig. 2)^5^. In purine biosynthesis, two glutamines are used to generate inosine monophosphate (IMP), a precursor of both adenosine monophosphate (AMP) and guanosine monophosphate (GMP). Then, one glutamine molecule is needed for the conversion of IMP to GMP^31^. In pyrimidine biosynthesis, one glutamine molecule is consumed by a carbamoyl phosphate synthetase enzyme (CPS1 or CPS2, which are localized in the mitochondria and cytosol, respectively). One more glutamine molecule is used to synthesize cytidine triphosphate (CTP) from uridine triphosphate (UTP)^31^. Interestingly, the first step in de novo pyrimidine biosynthesis mediated by CPSs occurs mainly in mitochondria via CPS1 in K-Ras/LKB1-mutant lung cancer cells^32^ (Fig. 2). Although cytosolic CPS2 can produce a cytosolic pool of carbamoyl phosphate, CPS1 is a major rate-limiting enzyme in pyrimidine biosynthesis using nitrogen released via mitochondrial glutaminolysis^32^.

In addition, glutamine can support nucleotide synthesis via other pathways. Aspartate, which is derived from the transamination of glutamine to form glutamate, participates in pyrimidine and purine biosynthesis^28^. Thus, exogenous aspartate can restore cell cycle arrest caused by glutamine deprivation^33^. Moreover, glutamine-induced activation of mTORC1 results in the phosphorylation of the enzyme complex called carbamoyl phosphate synthetase 2, aspartate transcarbamylase, and dihydroorotase (CAD), which catalyzes the condensation reaction converting glutamine-derived nitrogen into the pyrimidine precursor orotate^34,35^. Notably, increased expression of the transcription factor MYC, which is strongly associated with glutamine metabolism, induces the expression of several key enzymes in nucleotide biosynthesis, including phosphoribosyl pyrophosphate amidotransferase (PPAT)^36^. PPAT transfers glutamine-derived nitrogen to 5-phosphoribosyl pyrophosphate (PRPP), and this step is considered the initial step in purine biosynthesis^37^. In pancreatic cancer cells, oncogenic K-Ras maintains the nucleotide pool via the MAPK-dependent signaling pathway, leading to MYC upregulation, and the use of MEK inhibitors reduces the incorporation of glutamine-derived nitrogen into purine nucleotides^38^. Collectively, these studies describe a mechanism by which glutamine-derived nitrogen is essential for the rapid proliferation of cancer cells corresponding to an urgent need for nucleotide biosynthesis.

### NEAAs synthesized from glutamine

**Fig. 3:**
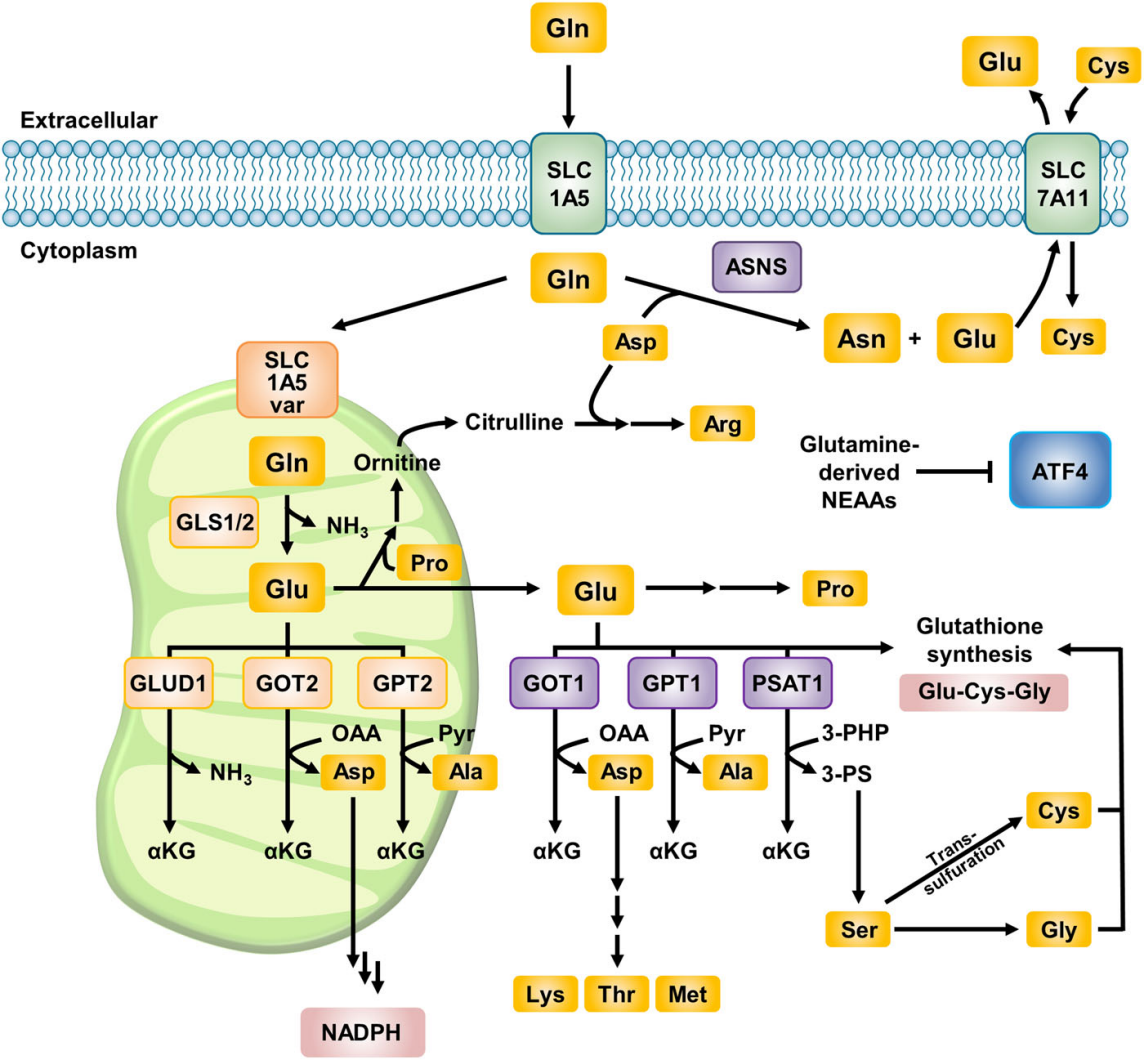
NEAAs synthesized from glutamine. Intracellular glutamine is converted into diverse NEAAs and supports protein translation and amino acid signaling. Glutamine-derived glutamate plays a central role as a substrate for several aminotransferases producing aspartate, alanine, proline, arginine, serine, cysteine, and glycine. ASNS directly utilizes cytosolic glutamine to synthesize Asn, which plays a distinct role in glutamine-related metabolism. Collectively, glutamine-derived NEAAs suppress ATF4, which is a master transcriptional regulator stimulated under stress conditions. NEAAs nonessential amino acids, GLS glutaminase, GLUD glutamate dehydrogenase, GOT glutamic-oxaloacetic transaminase, GPT glutamic-pyruvate transaminase, PSAT phosphoserine aminotransferase, ATF activating transcription factor, ASNS asparagine synthetase, Gln glutamine, Glu glutamate, Pro proline, Asp aspartate, Ala alanine, Ser serine, Gly glycine, Cys cystine, Asn asparagine, Lys lysine, Thr threonine, Met methionine, aKG α-ketoglutarate, OAA oxaloacetate, Pyr pyruvate, PHP phosphohydroxypyruvate, PS phosphoserine.

Although glutamine has been considered an NEAA that is synthesized endogenously, most cancer cells cannot proliferate or survive in a medium that does not contain glutamine^5^. This inability is probably due to the function of glutamine metabolism, which provides both carbon and nitrogen for cellular biogenesis. Glutamine-derived carbon is an important substrate that supports the TCA cycle and the synthesis of glutathione. In addition, nitrogen derived from glutamine is required for the biosynthesis of molecules such as nucleotides, glucosamine, and NEAAs^39^. Notably, among NEAAs, the generation of glutamate and asparagine is directly dependent on glutamine (Fig. 3).

### Glutamate

Glutamate plays a central role in NEAA metabolism because it is crucial for the biosynthesis of alanine, aspartate, proline, and serine, which are in turn used for the biosynthesis of asparagine, arginine, cysteine, and glycine (Fig. 3). Glutamate is converted to α-KG both via GLUD1, generating glutamate-derived nitrogen as ammonia, and via aminotransferases, which transfer nitrogen from glutamate to α-KG to produce other NEAAs. Glutamate consumption by aminotransferases to generate NEAAs has also been indicated to be required for tumor growth in diverse cancer types^29,40–42^.

Although glutamate is the major downstream product of glutamine, glutamate supplementation during glutamine deprivation cannot rescue the impaired cell growth or mitochondrial respiration^16,43–45^, indicating that mitochondrial GLS-catalyzed cleavage of the gamma-nitrogen of glutamine is essential for glutaminolysis. A possible reason for this requirement is the charge difference between glutamine and glutamate. Glutamine is a neutral amino acid and thus does not induce a negative charge burden in the mitochondrial matrix, which is already more negatively charged than the cytosol. Glutamate, however, is a negatively charged amino acid, and most cancer cells export—instead of import—glutamate^46^. Glutamate efflux is more crucial when NRF2 is activated. In cells with NRF2 activation, most glutamate is secreted, and cystine is imported by the SLC7A11 (xCT) antiporter mechanism^47^ (Fig. 3).

Glutamate is also utilized to synthesize the antioxidant glutathione^4^. The first reaction in glutathione synthesis is the ligation of glutamate and cysteine catalyzed by glutamate-cysteine ligase (GCL). Next, glycine is added by glutathione synthetase (GSS). Additionally, glutamate can be converted to glycine through a transamination reaction catalyzed by phosphoserine aminotransferase (PSAT1) into phosphoserine (3-PS) and α-KG. Phosphoserine is subsequently converted to glycine via serine hydroxymethyltransferase (SHMT) (Fig. 3). In cancer cells, the use of glutamate-derived nitrogen for NEAA production may be favored in various types of cancer cells to preserve nitrogen for anabolic reactions^48^ and may prevent apoptosis induced by ATF4 activation upon glutamine deprivation^6^.

### Asparagine

Asparagine can be synthesized de novo from glutamine via asparagine synthetase (ASNS). Interestingly, asparagine was reported to be able to rescue cancer cells from glutamine deprivation-induced apoptosis^43^. This finding is surprising because asparagine supplementation does not restore the levels of other NEAAs (alanine, proline, and glutamate) or any TCA cycle intermediates (α-KG, malate, and fumarate). Instead, asparagine supplementation enhances the expression of glutamine synthetase (GLUL) and increases intracellular glutamine usage via glutaminolysis, resulting in the recovery of global protein translation that is blocked by glutamine deprivation^45^. These studies suggest that most glutamine-dependent protein translation activities can still proceed under asparagine supplementation in a glutamine-deprived environment, although the exact mechanism is still unknown. Furthermore, studies performed in endothelial cells, Kaposi’s sarcoma-associated herpesvirus (KSHV)-transformed cancer cells and several normal fibroblast or epithelial cell lines reported a similar effect of asparagine on supporting cell survival and protein translation after glutamine deprivation^44,49,50^. Interestingly, high intracellular asparagine levels have recently been identified to be essential for breast cancer metastasis^51^. This study suggested that l-asparaginase treatment alone can reduce the incidence of breast cancer metastasis to the lung without affecting primary tumor growth. Although the clinical effect of l-asparaginase clearly indicates that asparagine is crucial for tumor survival and metastasis^52^, the importance of asparagine beyond protein synthesis and the mechanism by which asparagine supplementation enhances glutamine-associated metabolism are less well understood. Recently, asparagine has been reported to function as an exchange factor needed for the uptake of other amino acids that are required for mTORC1 activation^53^ and for enhanced nucleotide biosynthesis under mitochondrial electron chain transport system impairment^54^. Further investigation is needed to explain the considerable mechanistic importance of asparagine in cancer metabolism.

### Redox control of glutamine

A low level of reactive oxygen species (ROS) activates tumorigenic growth signaling; however, when the level exceeds the cellular redox capacity, ROS can damage macromolecules such as proteins, lipids and nucleotides^55^. Recent studies suggest that cancer cells are under increased oxidative stress caused by oncogenic transformation, leading to metabolic alterations that result in ROS production^56^. Under these conditions, glutamine metabolism becomes essential for maintaining cellular redox homeostasis by harnessing enhanced ROS levels. The metabolic pathway by which glutamine mitigates ROS is the glutathione synthesis pathway^57^ (Fig. 3). Glutathione is a tripeptide (Glu–Cys–Gly) that deactivates peroxide-free radicals. Glutamine is considered the rate-limiting factor in glutathione synthesis^58,59^. Indeed, experiments using uniformly labeled ^13^C-glutamine showed that glutathione was enriched with five ^13^C atoms in glutathione, suggesting that glutamine is the major source of glutathione^16,57,60^. As shown in Fig. 3, glutamine is a direct fuel for the use of glutathione as a source of glutamate and is indirectly responsible for cystine uptake via the xCT antiporter system, which takes up cystine and simultaneously secretes glutamate^61^. Consistent with this observation, glutamine starvation has been associated with impaired uptake of cystine through xCT and decreased intracellular glutathione levels^62^. Furthermore, cells in several types of cancers are characterized by significant enhancement of glutathione biosynthesis, and this metabolic vulnerability has been targeted to sensitize these cancer cells to ROS-induced drugs^63^.

Glutathione can be recovered from its oxidized form, accompanied by the conversion of NADPH to NADP^+^. In pancreatic cancer cells, glutamine supports the production of NADPH via a noncanonical metabolic pathway^29^, and the mitochondrial glutamine transporter is strongly associated with glutaminolysis-induced NADPH generation^16^. In addition, IDH1-dependent reductive glutamine metabolism produces NADPH, which decreases mitochondrial ROS during anchorage-independent growth^64^. In summary, glutamine maintains cellular redox homeostasis by supplying fuels for glutathione synthesis and endowing reducing power in the form of NADPH for sustaining tumor growth.

### Control of glutamine metabolism by hypoxia

**Fig. 4:**
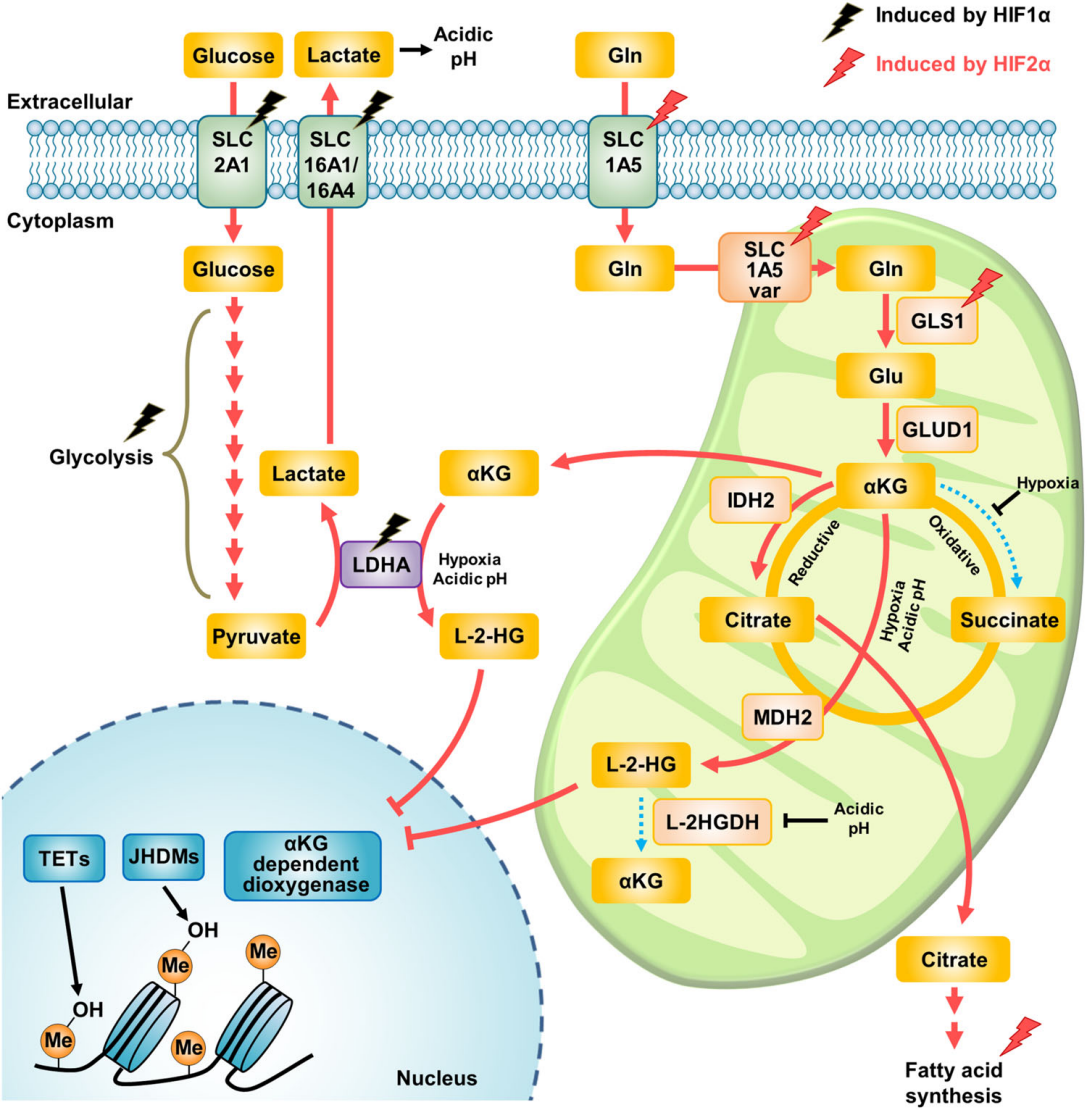
Control of glutamine metabolism by hypoxia. Hypoxia stabilizes HIF-α proteins such as HIF-1α and HIF-2α. HIF-1α enhances glucose uptake and increases the level of glycolytic enzymes. Under hypoxic conditions, most glucose-derived pyruvate is converted into lactate via LDHA and exported to the extracellular space through the lactate transporters SLC16A1 and SLC16A4. Under these conditions, HIF-2α-mediated glutaminolysis becomes essential to support the adaptation to hypoxia, altering the metabolic fate of glutamine via reductive carboxylation to generate citrate. Then, citrate participates in fatty acid synthesis in the cytosol, which is also activated by stabilized HIF-2α. Hypoxia-induced acidic pH also plays a crucial role in the production of L-2-HG by affecting the substrate affinities of LDHA and MDH. Next, L-2-HG can control DNA or histone methylation levels by regulating α-KG-dependent dioxygenases. HIF hypoxia-inducible factor, GLS glutaminase, GLUD glutamate dehydrogenase, IDH isocitrate dehydrogenase, MDH malate dehydrogenase, L-2HGDH L-2-hydroxyglutarate dehydrogenase, LDHA lactate dehydrogenase, TETs ten-eleven translocation enzymes, JHDMs JmjC domain-containing histone demethylases, Gln glutamine, Glu glutamate, α-KG α-ketoglutarate, L-2-HG L-2-hydroxyglutarate, Me methylation.

Hypoxic conditions promote the uptake of glutamine by increasing the levels of glutamine transporters such as SLC1A5, the SLC1A5 variant, and SLC38A2^16,65^ and switch the fate of glutamine from the oxidative pathway into the reductive carboxylation pathway^24^. This metabolic adaptation is critical because of the reduced entry of pyruvate into the TCA cycle by activated PDK1 and the increased lactate secretion in hypoxia^66^. Via this metabolic adaptation, cells can continually generate TCA metabolites, such as α-KG and citrate, which are converted to cytosolic acetyl-CoA for lipid biosynthesis (Fig. 4).

HIF-α is the most well-known transcription factor activated in hypoxia. HIF-1α is activated due to blockade of its degradation pathway mediated by low oxygen levels, thereby increasing the expression of target genes, including those encoding glycolytic enzymes and glucose transporters, and increasing lactate secretion^67^. Although HIF-2α has biochemical characteristics similar to those of HIF-1α, the metabolic role of HIF-2α in a low-oxygen environment is relatively unknown^68^. Recently, hypoxia-induced expression of the SLC1A5 variant was shown to be mediated by HIF-2α and to lead to metabolic reprogramming toward glutamine metabolism in pancreatic cancer cells^16^. Given that HIF-2α is an important transcription factor in cancer progression and leads to poor prognosis^69,70^, these findings suggest that targeting HIF-2α might be an effective therapeutic strategy by inhibiting glutamine metabolism in these notorious cancers. Furthermore, long-term exposure of cancer cells to acidic extracellular conditions induces metabolic reprogramming toward glutamine metabolism via HIF-2α activity^71^. In addition, extracellular lactate stabilizes HIF-2α, and HIF-2α then transactivates MYC, increasing the levels of glutamine transporters and GLS1, in turn resulting in increased glutamine catabolism^72^. These findings indicate that just as HIF-1α generally affects glucose metabolism in hypoxia, HIF-2α also plays a distinct role in glutamine metabolism to promote metabolic adaptation in hypoxia (Fig. 4).

Fatty acid synthesis is an anabolic process that uses cytosolic citrate to produce acetyl-CoA^73^. Glutamine acts as an alternative fuel for fatty acid synthesis, supplying citrate via mitochondrial reductive carboxylation, especially under hypoxic conditions^74,75^. In the context of constitutive HIF-2α stabilization^75^ or a defective mitochondrial electron transport chain^76^, glutamine-derived α-KG is reductively carboxylated through the consumption of NADPH by IDH2 to generate citrate. Next, mitochondrial citrate is transported across the inner mitochondrial membrane via a citrate carrier (CIC or SLC25A1) to support fatty acid synthesis for tumor progression in hypoxia^73^ (Fig. 4). This mechanism is very important in clear cell renal cell carcinoma (ccRCC) in which HIF-2α signaling is constitutively activated and intracellular lipid droplets are abundant. Fatty acid synthesis induced by HIF-2α is crucial for cell viability in ccRCC by sustaining endoplasmic reticulum (ER) homeostasis^77^. Furthermore, HIF-2α represses the transcription of carnitine palmitoyltransferase 1A (CPT1A), which is responsible for mitochondrial β-oxidation by transporting fatty acids and results in lipid deposition^78^. Indeed, recent studies have shown that HIF-2α can be targeted by selective inhibitors and have indicated that these molecules effectively suppress cancer cell growth and tumor angiogenesis characteristics in ccRCC^79–82^. Thus, HIF-2α-induced fatty acid synthesis using glutamine-derived citrate can be therapeutically targeted in several cancers, especially ccRCC.

In several cancers, glutamine metabolism is closely related to hypoxia-induced chemoresistance^83^. For example, glutamine depletion has been shown to abolish hypoxia-induced chemoresistance in cholangiocarcinoma. Impairing glutamine metabolism also induces sensitivity in gemcitabine-resistant pancreatic cancer cells^16,84,85^. This bolstered chemoresistance in cancer cells is partially supported by glutathione synthesis via glutaminolysis^86^. Given the importance of glucose and glutamine metabolism in pancreatic cancer cells, it is not surprising that gemcitabine resistance is closely associated with metabolic status, including cellular glucose and glutamine levels. Hypoxia increases the deoxycytidine triphosphate (dCTP) level through the pentose phosphate pathway (PPP) via glucose metabolism and results in resistance to gemcitabine, a dCTP analog^87^. Furthermore, redox modulation augmented by increased glutathione synthesis from glutamine was reported to be the mechanism of resistance to gemcitabine in pancreatic cancer cells^16^. Consistent with these findings, while NRF2 induces chemoresistance in KRAS-driven cancers, suppressing glutamine metabolism leads to weakened chemoresistance in these cancer cells^85^. These studies suggest that targeting glutamine metabolism can be an effective cancer treatment strategy when combined with conventional anticancer chemotherapy.

Under hypoxic conditions, L-2-hydroxyglutarate (L-2-HG) was proven to be generated by lactate dehydrogenase A (LDHA) and malate dehydrogenase (MDH)^88,89^. Under normal physiological conditions, LDHA catalyzes the conversion of pyruvate to lactate. However, under hypoxic conditions, LDHA can produce L-2-HG. The cellular metabolic alteration of increased L-2-HG levels contributes to the regulation of histone and DNA methylation levels by inhibiting epigenetic modification enzymes that use α-ketoacid as a cofactor. These events mitigate cellular reductive stress by suppressing key metabolic pathways, indicating a crucial role of L-2-HG. Acidic pH has also been reported to induce L-2-HG production via the promiscuous activity of LDHA and MDH enzymes. Acidic pH impairs the activity of the mitochondrial L-2-HG removal enzyme L-2-hydroxyglutarate dehydrogenase (L2HGDH) and enhances the protein stabilization of HIF-1α, leading to its escape from the degradation pathway^90^. In addition, L-2-HG accumulation in an acidic pH environment has been reported to result in HIF-1α stabilization in normoxia^91^ (Fig. 4).

Homozygous L2HGDH mutations in germline transmission cause a disease named 2-hydroxyglutaric aciduria (L-2-HGA)^92^. L-2-HGA is an autosomal recessive encephalopathy with an onset in childhood that causes developmental delays, epilepsy and cerebellar ataxia, the traditional clinical signs of this condition. Interestingly, patients with L-2-HGA are affected by tumors, including brain tumors^93^, bone tumors^94^, and nephroblastoma (Wilms tumor)^95^. Furthermore, increased L-2-HG levels caused by reduced expression of L2HGDH have been reported in renal cancer^96^. These studies indicate an oncogenic effect of L-2-HG and the association of L-2-HG with tumorigenesis under hypoxic conditions.

### Control of epigenetic changes by glutamine

The metabolic state constitutes a fundamental component of chromatin modification and genome regulation^97^. As metabolites are the substrates used to generate chromatin modifications, including methylation and acetylation modifications of histones, a complicated interaction exists between metabolism and epigenetics. In particular, glutamine-derived α-KG has been implicated in regulating cellular histone and DNA methylation levels^98^.

**Fig. 5:**
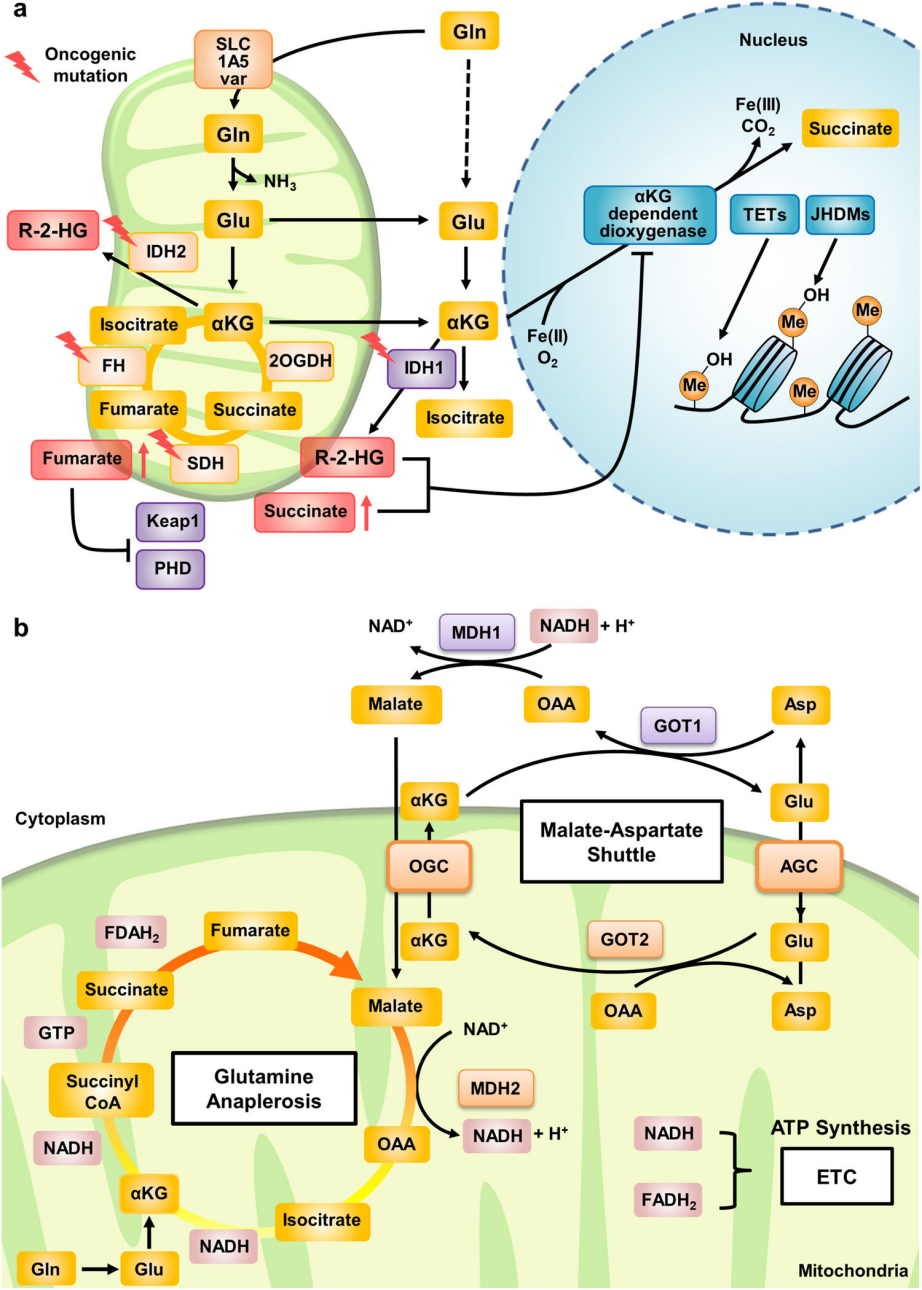
Glutamine oncometabolites and energy production from glutamine. **a** Several mutations in enzymes in the glutaminolysis pathway are responsible for the production of oncometabolites. Mutation of IDH1 and IDH2 produces R-2-HG from α-KG, which, when accumulated, leads to the inhibition of dioxygenases, in turn leading to the activation of TET and JHDM enzymes inside the nucleus. Mutation of SDH arrests the TCA cycle, resulting in an increase in the succinate concentration. A high concentration of succinate has an effect similar to the oncometabolite effect of R-2-HG. Additionally, impaired function of FH prevents further metabolism of fumarate, leading to its accumulation. FH impairment inhibits the function of Keap1 and PHD, which stimulates the transcription of protooncogenes. Gln glutamine, Glu glutamate, α-KG α-ketoglutarate, IDH isocitrate dehydrogenase, 2OGDH 2-oxoglutarate dehydrogenase, SDH succinate dehydrogenase, FH fumarate hydratase, R-2-HG R-2-hydroxyglutarate, Keap1 Kelch-like ECH-associated protein 1, PHD prolyl hydroxylase, TETs ten-eleven translocation enzymes, JHDMs JmjC domain-containing histone demethylases, Me methylation. **b**. Glutamine anaplerosis is a key mitochondrial metabolic pathway for cancer cell growth and survival. Influx of glutamine-derived α-KG into the TCA cycle replenishes the intermediates and consequently generates NADH, FADH_2_, and GTP. The generated GTP can be readily converted to an equal amount of ATP. Additionally, glutamate and α-KG produced via glutaminolysis participate in the malate-aspartate shuttle, promoting the transport of NADH from the cytosol into mitochondria. Elevated mitochondrial NADH and FADH_2_ levels collectively contribute to enhanced ATP production via OXPHOS through the ETC. Gln glutamine, Glu glutamate, Asp aspartate, αKG α-ketoglutarate, GOT1/2 glutamic-oxaloacetic transaminase 1/2, MDH1/2 malate dehydrogenase 1/2, OAA oxaloacetate, OGC 2-oxoglutarate carrier, AGC aspartate-glutamate carrier, ETC electron transport chain.

α-KG, also named 2-oxoglutarate, is a cofactor for 2-oxoglutarate-dependent dioxygenases (2-OGDDs), which catalyze hydroxylation reactions on diverse substrates. The activities of 2-OGDDs are affected by the intracellular level of α-KG, succinate, fumarate, or 2-HG. These hydroxylation reactions also require Fe^2+^ as a cofactor, O_2_ as a cosubstrate and ascorbic acid (vitamin C) as a reductase, which restore the activity of 2-OGDD enzymes (Fig. 5a). Among 2-OGDDs, Jumonji C domain-containing histone demethylases and ten-eleven translocation (TET) family DNA demethylases are major enzymes that induce epigenetic modifications using glutamine-derived α-KG. In these reactions, α-KG is oxidized to succinate, and increasing levels of succinate can suppress the progression of α-KG-dependent histone or DNA demethylase reactions^98^.

In cancer cells, mutations in succinate dehydrogenase subunit B (SDHB) cause susceptibility to familial pheochromocytoma^99^ and familial paraganglioma^100^ as well as gastrointestinal stromal tumors^101^. An increased ratio of succinate to α-KG in cancers resulting from impaired succinate dehydrogenase (SDH) activity is related to pervasive DNA hypermethylation, which contributes to the downregulation of key genes implicated in cell differentiation and cancer stages^102^. Moreover, the core region of solid tumors exhibits a deficiency of glutamine compared with other amino acids. This severe glutamine deprivation leads to dramatic histone hypermethylation due to decreased α-KG levels subsequent to decreased activity of Jumonji domain-containing histone demethylases and results in cancer cell dedifferentiation and resistance to BRAF inhibitors^103^.

In addition to its role in cancer cells, α-KG supports the self-renewal of naive murine embryonic stem cells (mESCs) by promoting histone and DNA demethylation^104^. In addition, at later stages of pluripotency, α-KG derived from glutamine can promote early differentiation, suggesting that the stage of cellular maturity can alter the effect of α-KG^105^. Furthermore, PSAT1 regulates changes in the level of glutamine-derived α-KG, which controls mESC pluripotency and differentiation^106^. These reports suggest that α-KG generated via glutaminolysis is closely related to the cellular decisions that characterize stem cells. In skeletal stem cells (SSCs), GLS and glutamine metabolism are required for the regulation of osteoblast and adipocyte specification and physiological bone formation^107^. In macrophage cells, α-KG produced via glutaminolysis promotes M2 activation via Jmjd3-dependent metabolic and epigenetic reprogramming^108^.

In T cell activation, glutamine deprivation has been shown to alter the activation of naive CD4^+^ T cells and result in their differentiation into forkhead box P3-positive (Foxp3^+^) regulatory T (T_reg_) cells, which have suppressor functions^109^. Recently, glutamine metabolism has been shown to be linked to white adipose tissue (WAT) inflammation in obesity^110^. The researchers discovered that glutamine metabolism is impaired in the obese state, leading to increased chromatin O-GlcNAcylation and activation of genes in proinflammatory pathways.

Collectively, glutamine-derived metabolites act as epigenetic modulators in a wide range of cell and tissue types, including various types of cancer cells, stem cells, immune cells, and even adipocytes. Considering that the SLC1A5 variant is an important regulator of the production of glutamine-derived α-KG^16^, confirming whether epigenetic regulation by glutamine-derived α-KG is affected by the SLC1A5 variant in cancer cells or stem cells is necessary (Fig. 5a).

### Glutamine and its oncometabolites

The discovery of R-2-hydroxyglutarate (R-2-HG) accumulation in several tumors encouraged investigators to initially establish the term “oncometabolite”^111^. Genetic and metabolic studies have further shown that metabolites such as succinate and fumarate, which are generated under normal physiological conditions, are associated with tumorigenesis in several cancer types. Interestingly, these metabolites were often found to be associated with glutamine metabolism^112^. In particular, the production of these oncometabolites was affected by the level of glutamine-derived α-KG. Although additional studies are needed, ample experimental data support the recognition of R-2-HG, succinate, and fumarate as oncometabolites.

### R-2-HG

Wild-type IDH1 and IDH2 catalyze the reaction by converting isocitrate and NADP^+^ into α-KG and CO_2_ with the concomitant generation of NADPH in the cytosol and mitochondrial matrix. However, mutant IDH enzymes convert α-KG into R-2-HG with the oxidation of NADPH into NADP^+^. Thus, various tumors, including glioma, secondary glioblastoma, and acute myeloid leukemia (AML), harboring heterozygous point mutations in the active sites of IDH1/2 show dramatic increases in the R-2-HG levels^111,113–115^. A high level of R-2-HG is sufficient to cause leukemia to arise from hematopoietic cells by maintaining their dedifferentiation and proliferation activities^116^. The role of R-2-HG as an oncometabolite has been implicated in epigenetic modifications through the inhibition of α-KG-dependent dioxygenases and demethylases, which has been assumed to be a driver of tumorigenesis^117,118^. In addition, dysregulated α-KG flux from normal reductive anabolism via the TCA cycle toward R-2-HG production has been associated with other metabolic flux impairments and disrupted redox balance^119,120^ (Fig. 5a).

Interestingly, the generation of R-2-HG from glutamine has been proven to occur rapidly in patient-derived chondrosarcoma cell lines harboring endogenous IDH mutations, indicating fundamental metabolic differences between cells that harbor IDH1/2 mutations and those that do not^121^. In this study, glutamine flux was directed toward the generation of R-2-HG in IDH1/2 mutant cells, and the kinetics of R-2-HG formation were proportionally of the same order of magnitude as those of glutamate or α-KG formation via glutaminolysis. Indeed, glutamine-derived R-2-HG accumulates and prevents the differentiation of myeloblasts, resulting in uncontrolled growth of blood cells^122^. After FDA approval of enasidenib, a first-in-class drug targeting cancer metabolism via inhibition of IDH2 activity, more studies were conducted with R-2-HG positioned as an oncometabolite. CB-839, a GLS inhibitor that blocks the conversion of glutamine into glutamate, reduced the production of R-2-HG in AML cell lines and patient tissues harboring IDH1/2 mutations^123^. As the importance of R-2-HG in boosting tumor initiation, proliferation and metastasis is emphasized, identifying whether metabolic enzymes or transporters associated with glutamine metabolism could be involved in the generation of R-2-HG is interesting.

### Succinate

The normally functioning SDH enzyme is localized in the inner mitochondrial membrane and plays a role in the electron transport chain as well as the conversion of succinate into fumarate. In 2008, mutation of SDH was discovered in cancers such as paraganglioma and pheochromocytoma cells^124^. Later, similar observations were made in gastrointestinal tumors, neuroblastomas, renal tumors, thyroid tumors, and testicular tumors^125,126^. Several research groups have focused on the mechanism that underlying the features of tumorigenesis and cancer cell survival in the setting of SDH mutations. As succinate accumulates via the inhibition of the 2-OGDD enzyme, epigenetic modification acts in the process of cell transformation into a hypermethylated phenotype^100^. Several studies have shown that SDH-deficient cells exhibit increased tumorigenesis and that this increase is reversed by the addition of α-KG, supporting the idea that succinate accumulation contributes to tumorigenesis through epigenetic modification^100^. Succinate-specific effects are initiated by epigenetic alterations through the inhibition of KDMs and the TET family 5mC hydroxylases, which induce the translation of tumorigenic genes (Fig. 5a). The other mechanism by which succinate supports tumorigenesis acts through the inhibition of hypoxia-inducible factor prolyl hydroxylase (PHD). PHD activates the pseudohypoxic response by stabilizing HIF-1α, which is a well-known tumorigenesis enhancer, and as a transcription factor, maintains the metabolic reprogramming of cancer cells to support their survival^127^. In addition to the tumorigenic effects of succinate accumulation, SDH5 mutation is the key driver supporting the acquisition of epithelial–mesenchymal transition (EMT) characteristics. The results of a clinical study further confirmed this observation by showing that patients with nonmetastatic lung cancer harbored loss-of-function mutations in SDH5^128^. The study of succinate as an oncometabolite has only recently begun, and more research needs to be conducted to completely understand its tumorigenic properties.

### Fumarate

Fumarate is another example of an oncometabolite produced by the action of fumarate hydratase on succinate. In 2001, mutation of fumarate hydratase leading to its inactivation was discovered in renal cell cancer^129^. Mutation of this enzyme leads to fumarate accumulation not only in skin cancer and uterine leiomyomas but also in breast, bladder, and Leydig cell tumors^130^. Further confirmation of fumarate as an oncometabolite was verified by experimental data showing that tumor cells lost their ability to invade and migrate when the function of fumarate hydratase was restored by an external expression vector^131^. In attempts to understand the cause of these effects, it was found that cells with high concentrations of fumarate display a phenotype of DNA hypermethylation. In addition, fumarate inhibits TET enzymes, which stimulate EMT, leading to cancer metastasis^131,132^. Similar to succinate, fumarate contributes to the inactivation of PHD, stabilizing HIF proteins to promote cell survival^133^ (Fig. 5a). In addition, accumulated fumarate can participate in different reactions of the addition of a succinate group to the thiol group of various proteins. For example, in hereditary leiomyomatosis and renal cell cancer (HLRCC), a high level of fumarate caused by genetic mutation of fumarate hydratase induces the succination of Kelch-like ECH-associated protein 1 (KEAP1) accompanied by the consumption of a fumarate molecule^134,135^. Endogenously, succinylated KEAP1 dissociates from the NRF2 protein to help cancer cells survive stress. High concentrations of fumarate bind to glutathione, augmenting ROS signaling and accumulation, as observed in not only in vitro models but also in vivo models^136,137^. Additionally, high levels of fumarate react with the cysteine group of mitochondrial aconitase-2 and iron-sulfur cluster binding protein-2, facilitating cellular metabolic adaptation to stresses^138^. The importance of fumarate hydratase mutation for cancer survival and growth is being studied in depth to completely understand the role of fumarate as a tumorigenic oncometabolite. This knowledge will aid in the complete comprehension of cancer metabolism.

### Glutamine-derived energy production

The influx of α-KG into the TCA cycle and its subsequent oxidization generates two molecules of NADH and one molecule of FADH_2_ from the series of reactions catalyzed by OGDH, SDH, and MDH. Additionally, when succinyl-CoA is converted to succinate by succinate thiokinase, one molecule of GTP is generated, which can be readily converted to ATP by nucleoside-diphosphate kinase (NDPK). NADH and FADH2 produced via glutaminolysis are then fed into the electron transport chain to create the electrochemical gradient necessary for ATP production via oxidative phosphorylation^139,140^ (Fig. 5b). Correspondingly, in K-Ras mutant cells, the oxygen consumption rate and ATP generation are enhanced by glutamine, contributing to tumorigenesis^55^. Moreover, after the activation of K-Ras and Akt in transformed cells, 60% of the total FADH_2_ and NADH_2_ are synthesized from glutamine, while only 30% is derived from glucose^140^. Additionally, the level of the mitochondrial glutamine transporter controls the cellular ATP level stimulated by glutamine, suggesting that glutamine is an important energy source via mitochondrial glutaminolysis^16^. Collectively, these observations indicate that anaplerotic glutamine metabolism is highly responsible for energy generation in cancer cells.

Additionally, NADH can be generated by fatty acid oxidation (FAO) in the cytoplasm in tissues with high energy demand, such as cardiac muscle tissues, as well as in cancer cells^141^. Recent studies have suggested that in cancer cells with elevated cytosolic NADH levels, the malate-aspartate shuttle (MAS) actively takes up NADH to produce ATP in mitochondria through the electron transport chain^142^. The MAS comprises MDH1/2, GOT1/2, the malate-α-KG antiporter and the glutamate-aspartate antiporter, which exchanges mitochondrial α-KG for cytosolic malate that is synthesized from oxaloacetic acid (OAA) by cytosolic MDH (Fig. 5b). Glutamate and α-KG serve as important exchangers in the MAS, and since GLS1 knockdown significantly suppresses NADH and ATP production in cancer cells^143^, the supply of glutamate and α-KG for the induction of MAS activity is evidently critical for ATP production in cancer cells (Fig. 5b).

### Glutamine metabolism upon cellular stresses

Glutamine is the most abundant amino acid in the blood. During cellular stress, such as nutrient starvation and catabolic stress after trauma, surgery, infection, sepsis, or cancer cachexia, blood glutamine levels are severely decreased^144^. Under these conditions, several studies have reported that glutamine supplementation can offer a therapeutic approach for these critical illnesses^145–147^. Glutamine has been considered an immunomodulatory amino acid in several disease states, yet the mechanisms underlying the therapeutic effects of glutamine supplementation in critical illness remain poorly understood. Conceivably, glutamine could exert its beneficial effects by producing glutathione for redox homeostasis, maintaining nitrogen balance, or other functions in immune cells^2^.

Consistent with the importance of glutamine in stressful situations, glutamine deprivation induces cellular stress. Upon glutamine starvation, p53 activity is induced and can help cancer cells adapt to nutrient starvation through diverse mechanisms^148^. Recently, SLC1A3, as a crucial effector of p53, has been shown to support cell survival and growth in the absence of glutamine^149^. Under DNA damage such as radiation, glutamine is conditionally essential to support the synthesis of nucleotides and redox homeostasis. It has recently been demonstrated that radioresistant cancer cells reprogram metabolic flux toward glutamine anabolism. Under these conditions, cancer cells highly express glutamine synthetase, facilitating cancer cell growth under radiation stress^150^. Moreover, evidence has shown that during the DNA damage response, normal cells show a decrease in glutaminolysis controlled by SIRT4 protein suppressing GLUD1. In the absence of SIRT4, a failure to undergo cell cycle arrest induced by DNA damage causes a delay in DNA repair and increased chromosomal instability, suggesting a tumor suppressor effect of SIRT4^151^.

Numerous studies have described the presence of alternative adaptive pathways upon the perturbation of glutamine metabolism. For instance, a recent study has shown that GLS1 inhibition induces an increase in mitochondrial glutamate-pyruvate transaminase 2 (GPT2) to assist in TCA cycle anaplerosis for sustaining cancer cell growth and survival^152^. Of note, GLS1 inhibition causes an elevation of the ROS level and induces GPT2 expression via ATF4, which again implies the importance of ATF4-mediated metabolic adaption during glutamine starvation.

Additionally, metabolic profiling has revealed that suppression of GLS1 induces a compensatory anaplerotic mechanism via pyruvate carboxylase (PC), which allows the release of a glutamine-independent supply of TCA intermediates by catalyzing the transformation of pyruvate to oxaloacetate^153^. This PC-mediated alternative anaplerosis is considered important in specific types of cancers, including liver cancers and glioblastoma, for maintaining biosynthesis and redox homeostasis^154–156^. Collectively, cancer glutamine metabolism shows extraordinary flexibility and is intertwined with diverse metabolic pathways.

## Metabolic reprogramming induced by glutamine metabolism

**Fig. 6:**
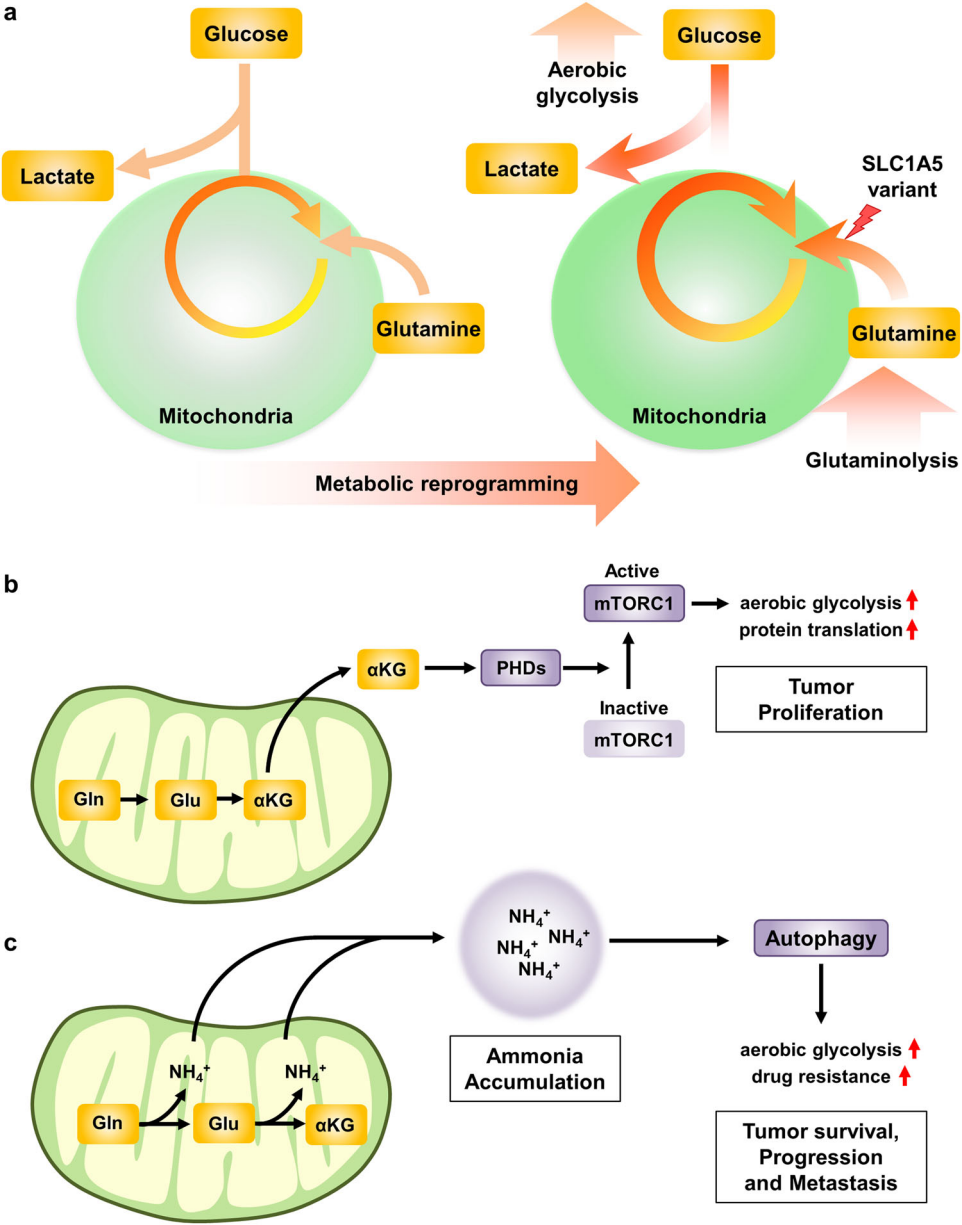
Metabolic reprogramming induced by glutamine metabolism. **a** Aerobic glycolysis is a hallmark of cancer metabolism. During this process, most glucose-derived pyruvate is secreted extracellularly as lactate, and glutamine becomes a conditionally essential amino acid. Glutaminolysis sustains mitochondrial function, supplying TCA cycle metabolites such as αKG and generating diverse biomolecules, including NEAAs, NADPH, and nucleotides. Increased glutamine flux into the mitochondrial matrix via the SLC1A5 variant can enhance glutaminolysis and lead to metabolic reprogramming toward enhanced aerobic glycolysis. **b** Glutamine-derived α-KG activates the mTORC1 signaling pathway, resulting in aerobic glycolysis and protein translation, which are crucial for tumor proliferation. **c** During glutaminolysis, ammonium ions are generated via a deamidation reaction catalyzed by glutaminase and glutamate dehydrogenase. Most ammonium ions are used as a nitrogen source for nucleotide biosynthesis and are disposed of via the urea cycle, but an excess of ammonium ions promotes autophagy. Augmented autophagy is associated with drug resistance by enhancing aerobic glycolysis and is involved in cancer cell survival, progression, and metastasis. Gln glutamine, Glu glutamate, α-KG a-ketoglutarate, PHD prolyl hydroxylase.

Unsurprisingly, glutamine metabolism plays a critical role in tumor progression since it not only supports mitochondrial oxidative phosphorylation but also supplies metabolic intermediates for the TCA cycle, glutathione synthesis, and NEAA synthesis and simultaneously produces NADPH^157–159^. Recently, glutamine was shown to be a major fuel for mitochondrial oxygen consumption in pancreatic cancer cells; in addition, the expression of the SLC1A5 variant affected the levels of metabolites derived from glucose metabolism, including lactate and ribulose-5-phosphate, the intermediate metabolites in the PPP^16^. Intriguingly, this study regarding elevated glutamine metabolism in cancer cells also showed that glutaminolysis could in turn reinforce metabolic reprogramming, thus implying that glutamine metabolism plays a crucial role in tumorigenesis and tumor progression^16^ (Fig. 6a). Indeed, the process of adaptation to glutamine deprivation weakens the response to hypoxia, which normally strongly induces the expression of glycolytic enzymes^83^.

As previously described, glutamine is metabolized by mitochondrial enzymes into α-KG, which serves as an important intermediate in the TCA cycle for anaplerosis. Furthermore, enhanced production of α-KG causes other critical effects, such as stimulation of the signaling pathways that support cell growth. α-KG induces mTORC1 activation by enhancing GTP loading of the RagB protein in a PHD-dependent manner, thus promoting cell growth^160,161^. Accordingly, high mTORC1 activity in cancer cells promotes aerobic glycolysis and drives glucose addiction^162,163^ (Fig. 6b). In addition, mTORC1 activation via glutaminolysis suppresses autophagy and the DNA damage response^164,165^. Therefore, enhanced glutaminolysis might eventually contribute to the initiation and progression of cancer by stimulating cell growth via the mTORC1 pathway and enhancing aerobic glycolysis while disrupting the proper elimination of misfolded proteins, damaged DNA and organelles through the inhibition of autophagy and the DNA damage response^166^.

Enhanced glutaminolysis in cancer cells ensures a stable supply of glutamate and α-KG via sequential deamination processes inside mitochondria. Notably, ammonia is simultaneously generated as a byproduct of glutamine deamination. Hence, the facilitation of glutaminolysis leads to the accumulation of excess ammonia within cells, and a high concentration of ammonia is a potent inducer of autophagy^167^ (Fig. 6c). Although mTORC1 activation hinders autophagy, evidence has shown that autophagy can be upregulated in tumors with mTORC1 hyperactivation^168^. Therefore, glutaminolysis can suppress autophagy by activating the mTORC1 pathway but, on the other hand, can stimulate autophagy in the context of excess ammonia production. The fundamental need for ammonia-mediated induction of autophagy in cancer cells could be due to the cytoprotective functions of this event that allow cells to survive under extreme conditions^166^. Specifically, autophagy suppresses anoikis induced by the detachment of cancer cells from the extracellular matrix (ECM) and hence promotes metastasis^169^. Furthermore, autophagy has been shown to promote glycolysis in hepatocellular carcinoma (HCC) cells by upregulating monocarboxylate transporter 1 (MCT1), which plays an important role in the transport of lactic acid^170^. Therefore, autophagy supports cancer progression and chemoresistance by allowing tumor cells to overcome both environmental and intracellular stress signals, including nutrient deprivation and chemotherapeutic cytotoxicities^167,171,172^ (Fig. 6c).

However, the connection between glutamine and metabolic remodeling in cancer from the perspective of glucose metabolic flux, the mTORC1 pathway and autophagy has yet to be fully explored. This link might partially be explained by considering that the intimately entwined glucose and glutamine metabolic pathways cooperatively support the TCA cycle and that glutamine performs diverse functions for maintaining cellular homeostasis. Collectively, in-depth investigation of the role of glutaminolysis in tumor progression might hold the key for decoding cancer metabolic plasticity.

## Crosstalk between glutamine metabolism and oncogenic signaling

**Fig. 7:**
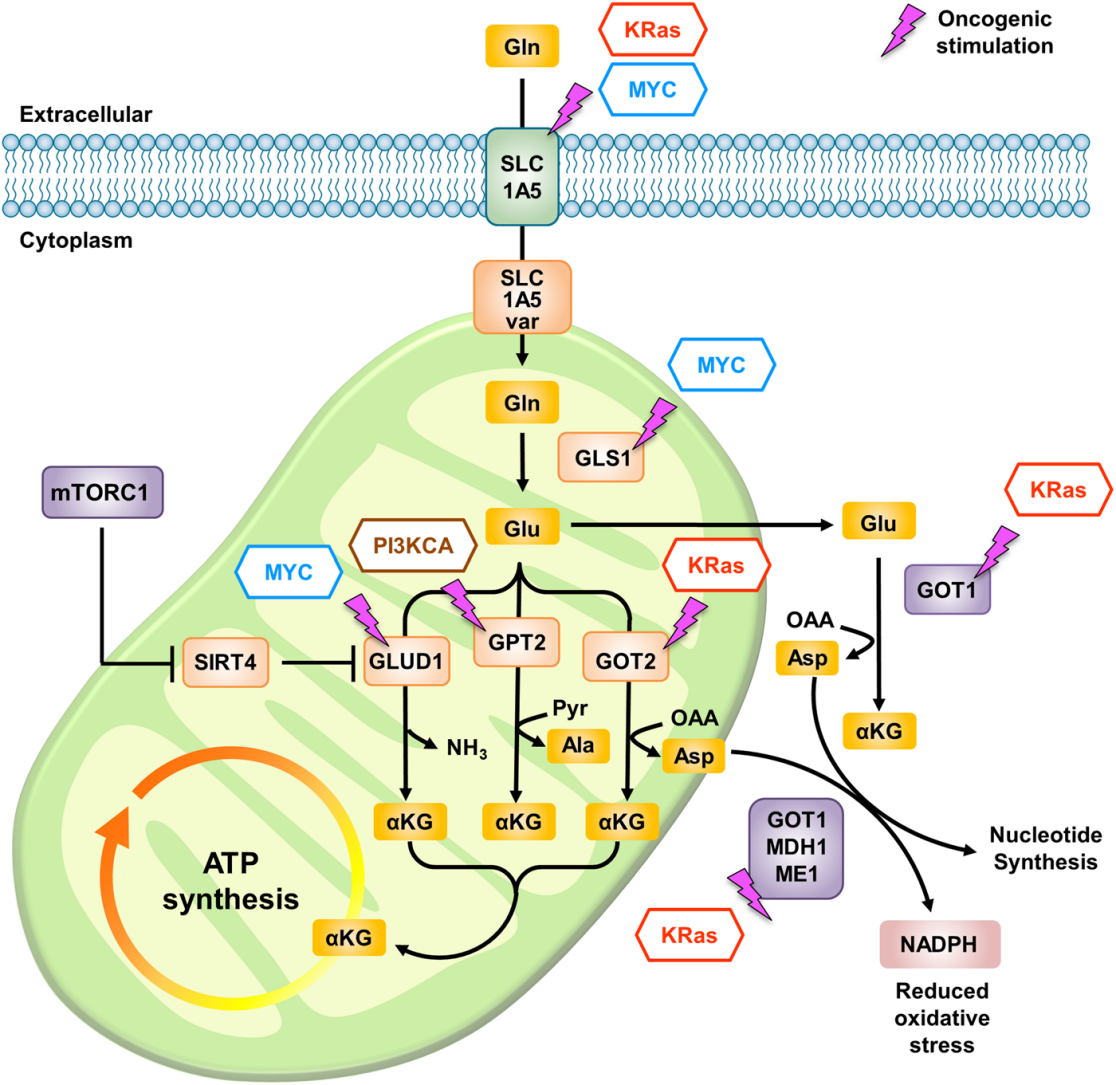
Oncogenic control of glutamine metabolism. Oncogenes such as MYC, K-Ras, and PI3KCA modulate cancer metabolic reprogramming, favoring cancer cell growth and survival partially via the promotion of glutamine metabolism. Glutamine uptake is enhanced in MYC- and K-Ras-driven cells in which the expression of the glutamine transporter SLC1A5 is upregulated. Deamination of glutamine to form glutamate in mitochondria is enhanced by MYC-mediated upregulation of GLS1. Conversion of glutamate into α-KG is mediated by GLUD1 or aminotransferases such as GOT1/2 and GPT2. The expression of these enzymes is upregulated in cancer cells with MYC-driven, K-Ras-driven, and PI3KCA-driven signaling activation. Gln glutamine, Glu glutamate, Ala alanine, Asp aspartate, α-KG α-ketoglutarate, GLS1 glutaminase 1, GLUD1 glutamate dehydrogenase 1, GOT1/2 glutamic-oxaloacetic transaminase 1/2, GPT1/2 glutamic-pyruvate transaminase 1/2, MDH1 malate dehydrogenase 1, ME1 malic enzyme 1.

The excessive proliferation exhibited by cancer cells demands a constant supply of fuels such as glucose and glutamine. Therefore, cancer cells orchestrate their metabolic pathways to coordinate their high demand for these nutrients. Metabolic reprogramming that promotes enhanced glutamine consumption in cancer cells is closely connected with dysregulation of oncogenes. Efforts have been undertaken to reveal the mechanism by which oncogenes modulate metabolic pathways that favor cancer cell growth^173^. Notably, cancer cells driven by oncogenic MYC, K-Ras, and PIK3CA require glutamine for their survival and display extensive anabolic utilization of glutamine^29,174,175^ (Fig. 7).

In cancer cells, genetic and epigenetic dysregulation of MYC expression and the loss of checkpoint components unleash the ability of MYC to promote cell growth, eventually leading to malignant transformation^176^. Oncogenic Myc stimulates mitochondrial glutaminolysis via transcriptional regulation of genes necessary for cellular glutamine catabolism^177^. MYC-driven cancer cells exhibit enhanced glutamine utilization accompanied by increased expression of key glutaminolysis enzymes, including GLS1/GLS2 and GLUD1^178–180^. Moreover, MYC upregulates the glutamine transporter SLC1A5 to facilitate glutamine uptake into cells^177^. MYC-dependent enhancement of mitochondrial glutaminolysis leads to the reprogramming of mitochondrial metabolism to accommodate the requirements for TCA cycle anaplerosis to sustain cellular viability and growth.

Similar to the situation in MYC-driven cancer cells, glutamine uptake is enhanced in K-Ras-driven cells via upregulation of SLC1A5^181^. Additionally, K-Ras-driven cells are characterized by increased expression of GOT1 and GOT2^182,183^. GOT1 and GOT2 catalyze the transamination reaction between oxaloacetate and glutamate to produce aspartate and α-KG. Significantly, enhanced transamination and aspartate synthesis in K-Ras-driven cancer cells are important in the promotion of nucleotide biosynthesis^184^ and maintenance of redox balance^29^.

Intriguingly, the glutamine-dependent checkpoint at late G1 phase in the cell cycle is dysregulated in K-Ras-driven cancer cells^185^. In normal cells, the cell cycle is tightly regulated by various checkpoints. Nutrient-dependent checkpoints regulate cell cycle passage through late G1 phase by sensing nutrient availability; glutamine is a particularly critical nutrient sensed in late G1 phase, and its deprivation causes cell cycle arrest at G1 phase^186^. Importantly, activation of K-Ras in cancer cells results in bypass of the late G1 glutamine-dependent checkpoint. Specifically, glutamine deprivation in K-Ras-driven cancer cells leads to growth arrest in S or G2/M phase instead of in G1 phase. Consistent with this observation, K-Ras sensitizes cells to glutamine deprivation, and K-Ras knockdown rescues cells from apoptosis induced by low glutamine levels^187^. Collectively, these findings indicate that enhanced glutamine metabolism and cell growth dysregulation are established in K-Ras-driven cancer cells to promote uncontrolled cell growth and to assist with glutamine acquisition and utilization for cell growth.

The PI3K signaling pathway is dysregulated in many tumors, and analyses have shown that PIK3CA is an oncogene that also contributes to tumor progression partially via metabolic reprogramming^188^. Oncogenic PIK3CA increases the dependency of cancer cells on glutamine by upregulating the expression of mitochondrial GPT2, which catalyzes the transamination reaction that converts glutamate and pyruvate into α-KG and alanine^175^. Thus, cells with PIK3CA mutations exhibit increased sensitivity to glutamine deprivation. Additionally, compared with wild-type cells, PIK3CA mutant colorectal cancer (CRC) cells exhibit elevated anaplerotic α-KG production and ATP generation from glutamine.

In addition to oncogenic regulators, there are some key upstream regulators of glutamine metabolism that are widely recognized for their pivotal role during tumorigenesis. mTORC1, which is well known for its function at the center of cancer metabolic reprogramming, promotes mitochondrial glutaminolysis via the migration of SIRT4-mediated inhibition of GLUD1^189^. Specifically, mTORC1 promotes proteasome-mediated destabilization of cAMP response element binding-2 (CREB2) to suppress transcription of SIRT4. Accordingly, loss of SIRT4 enhances glutamine-dependent proliferation and genomic instability, which simultaneously contribute to tumorigenesis^151^.

Furthermore, mTORC1 also acts as a downstream effector of glutamine. Glutamine itself, or after its conversion into α-KG, activates the mTORC1 pathway and participates in the growth signaling pathway. Evidence has shown that glutamine activates the mTORC1 pathway via Arf1 rather than via the Rag GTPase complex in MEFs^190^. According to another study, glutaminolysis increases the level of α-KG production, resulting in GTP loading of RagB and lysosomal translocation of the mTORC1 complex in human cancer cell lines^160^. It has been reported that cellular uptake of glutamine and its subsequent efflux in the presence of essential amino acids, including leucine, is the rate-determining step that activates mTORC1^191^. Moreover, glutamine also acts as a precursor for the synthesis of various NEAAs, including asparagine and arginine, implicated in mTORC1 activation^39^. Thus, cells have diverse mechanisms of mTORC1 activation for glutamine, and cancer cells efficiently utilize glutamine for mTORC1 pathway activation to drive unrestrained oncogenic growth.

## Targeting glutamine metabolism and therapeutic implications

Although the essential role of glutamine metabolism in cancer cells has been well demonstrated in vitro, the extent to which glutamine supports tumor growth and survival in vivo remains elusive. It has been reported that K-Ras-driven mouse lung tumors preferentially utilize glucose more than glutamine to supply carbon to the TCA cycle via pyruvate carboxylase^192^. Furthermore, human glioblastoma cells do not rely much on circulating glutamine for proliferation but rather more on glutamate to synthesize glutamine via glutamine synthetase to fuel purine biosynthesis^193^. Nevertheless, the specific metabolic importance of glutamine in tumorigenesis and tumor growth has also been reported^194–196^, and these studies have led many researchers to target glutamine metabolism for the treatment of cancer^8^. Throughout the discovery of agents targeting glutaminolysis, none have yet been used clinically^197^. A recent attempt focused on the inhibition of GLSs. GLS overexpression has been observed in different tumor cells, and these enzymes are found to function in the metabolic reprogramming of glutamine addiction in cancer^198^. Chemical agents targeting GLSs have been studied, and CB-839, 968, and BPTES have been found to exhibit tumor-specific antiproliferative effects^199^. Among these agents, CB-839 is the only one to proceed to clinical trials; however, its selectivity toward GLS1 and failure to inhibit the compensatory effect of GLS2 require in-depth study^14^. A recent study discovered a prodrug (JHU083) of the glutamine antagonist DON, which was designed to selectively become activated inside a tumor. The researchers showed that blocking glutamine metabolism through JHU083 not only suppressed tumor cell metabolism but also mitigated the tumor microenvironment, which is hostile to the immune response due to its hypoxic, acidic, and nutrient-depleted conditions, unleashing the natural antitumor T cell response. They also confirmed that concurrent treatment with JHU083 and anti-PD-1 checkpoint inhibitor improved the antitumor effects compared with anti-PD-1 treatment alone, suggesting the presence of metabolic plasticity between cancer cells and effector T cells, which could be exploited as a metabolic checkpoint for cancer immunotherapy^200^.

**Fig. 8:**
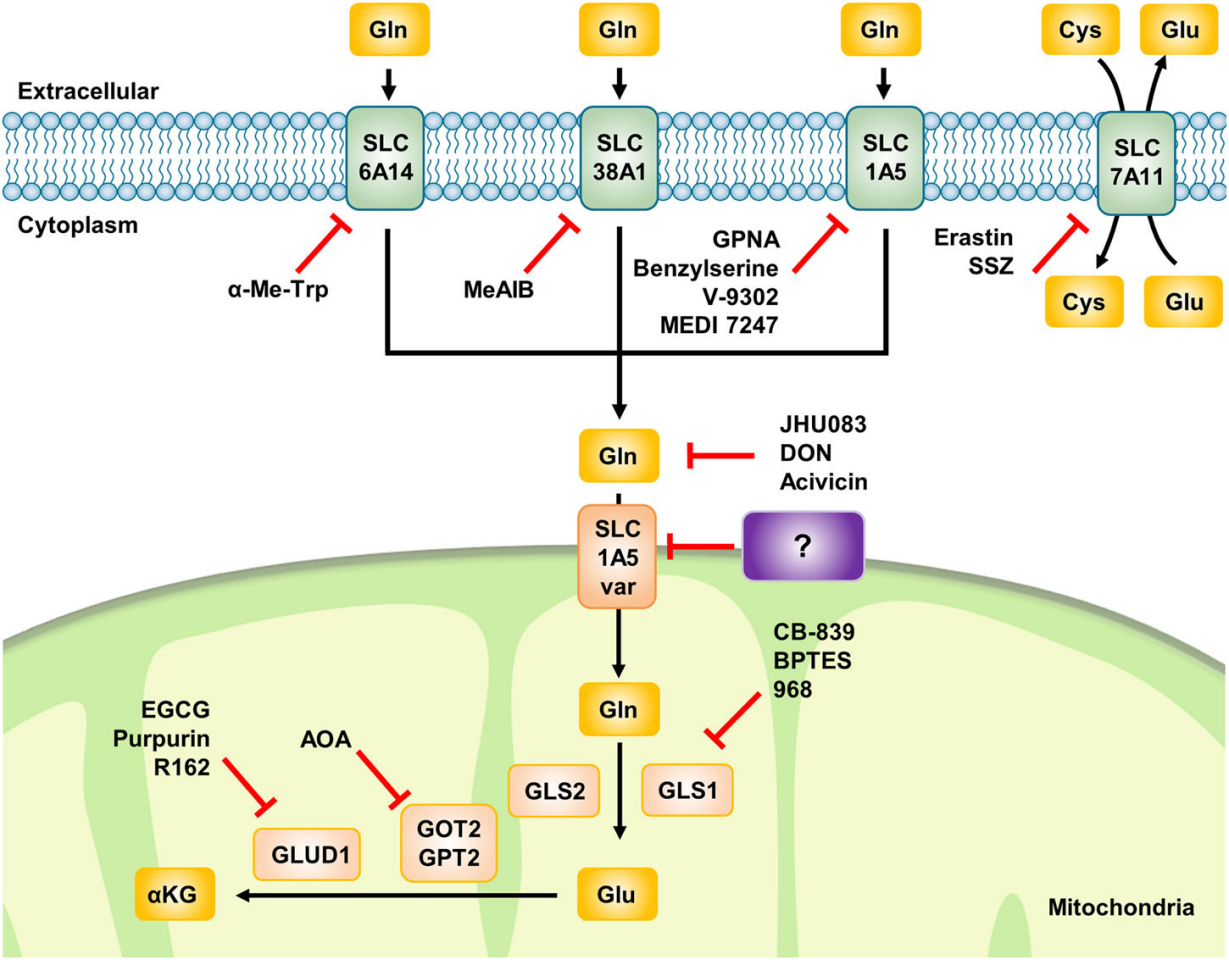
Inhibitors of glutamine transporters and glutaminolysis. For the principal inhibition of glutaminolysis, attempts have been made to target the amino acid transporters related to these pathways. SLC6A14 and SLC38A1 are inhibited by α-Me-Trp and MeIAB, respectively. The most intensely researched topic is inhibitors of SLC1A5, a major glutamine transporter, which include substrate analog competitive inhibitors such as GPNA, benzylserine, and V-9302 and the inhibitory antibody MEDI7247. Although they exhibit low potency, inhibitors of SLC7A11 include erastin and SSZ. Inhibitors of glutaminolytic enzymes are agents that target GLS1, GOT2, and GLUD1. CB-839, an agent in its 2nd clinical trial, inhibits GLS1 similarly to BPTES and 968. AOA inhibits GOT2 activity, and EGCG, purpurin, and R162 inactivate GLUD1. However, the SLC1A5 variant, the sole glutamine transporter discovered to date, is expected to be a much more effective target for cancer therapeutics than previously studied glutaminolysis inhibitors. Cys cysteine, Glu glutamate, α-KG α-ketoglutarate, GLS glutaminase, GOT2 glutamic-oxaloacetic transaminase 2, GPT2 glutamic-pyruvate transaminase 2, GLUD1 glutamate dehydrogenase 1, α-Me-Trp alpha-methyl-tryptophan, MeAIB methylaminoisobutyric acid, GPNA L-γ-glutamyl-p-nitroanilide, SSZ sulfasalazine, DON 6-diazo-5-oxo-l-norleucine, AOA aminooxyacetate, EGCG epigallocatechin-3-gallate.

The plasma membrane glutamine transporters SLC6A14, SLC7A11, and SLC38A1 have been targeted and found to be inhibited by erastin, α-Me-Trp, and MeAIB, respectively (Fig. 8). In addition, SLC1A5 was shown to have clinical importance, and it is considered the most critical plasma membrane glutamine transporter in cancer cells^201^. Many attempts have been made to explore the possibility that SLC1A5 suppression via small molecules might exert anticancer effects. As part of this effort, benzylserine and benzylcysteine were discovered in 2004 as the first substrate analog inhibitors of SLC1A5^202^. In an effort to improve the potency and efficacy of such inhibitors, some studies have discovered GPNA, which is widely used as a tool compound for suppressing SLC1A5^203^. Other studies have developed antibodies with high affinity for SLC1A5, which induce antibody-dependent cellular toxicity in gastric cancer models^204^. Recently, a potent inhibitor of SLC1A5, V-9302, has been reported to be effective in several cancer cell lines and in vivo tumor models^205^. However, other researchers have argued that controversial issues exist because GPNA also inhibits other glutamine transporters, such as SLC38A1, and V-9302 is effective even in SLC1A5 knockout models^206,207^. Hence, to date, no suitable compound has been identified to inhibit the plasma membrane glutamine transporter SLC1A5 with excellent sensitivity and specificity.

SLC1A5 might not be an appropriate target for suppressing glutamine uptake by cancer cells because it is not the only plasma membrane glutamine transporter, and its function would therefore be compensated by other redundant glutamine transporters such as SLC38A1 and SLC38A2. Thus, as the SLC1A5 variant is the only currently known glutamine transporter in the mitochondrial inner membrane^16^, targeting the SLC1A5 variant could be an effective strategy for selectively inhibiting glutamine metabolism in cancer cells (Fig. 8). Given the clinicopathological significance of SLC1A5^201^ and the observation that the level of the SLC1A5 variant is negatively correlated with prognosis in several cancer types^16^, targeting the SLC1A5 variant is a promising strategy to starve cancer cells and induce antitumor effects. Therefore, further studies on the development of selective inhibitors of the mitochondrial SLC1A5 variant are needed and should help to establish whether the level of the SLC1A5 variant is a predictive marker of glutamine dependency in cancer^21^.

## Conclusion

Although Otto Warburg characterized cancer metabolism by its enhanced glucose consumption and loss of mitochondrial function, many studies have shown that mitochondrial function in cancer cells is still robust and even enhanced. Moreover, glutamine has been discovered to be required for the maintenance of active mitochondrial function in cancer cells. Glutamine has historically been one of the most intensely investigated nutrients in cancer metabolism and is involved in various aspects of biosynthesis and bioenergetics, including NEAA production, epigenetic gene control, adaptation to hypoxic conditions, ATP synthesis, cell signaling, and tumorigenesis. In this review, we offer an updated overview of glutamine metabolism and discuss the reason for glutamine dependency in cell metabolism.

Certain types of cancer, including renal cell carcinoma, hematologic malignancies, glioblastoma, pancreatic cancer, and those reported to depend on HIF-2α, seem to depend on glutamine; hence, targeting glutamine metabolism may show therapeutic effects in these cancers. Moreover, metabolite transporters have recently been shown to be involved in tumorigenesis; for example, low levels of mitochondrial pyruvate carriers initiate colon cancer development^208^. Conversely, suppression of the SLC1A5 variant, a mitochondrial glutamine transporter, is sufficient to inhibit tumor growth by impairing glutamine metabolism in pancreatic cancer cells^16^. As the importance of subcellular metabolite transporters in controlling tumor initiation is poorly understood, it would be interesting to determine whether overexpression or knockout of these transporters is involved in tumorigenesis, metastasis, and immune modulation.

In conclusion, metabolic reliance on glutamine arises via the intrinsic functional diversity of glutamine, supporting macromolecule biosynthesis and reinforcing the TCA cycle. In the context of tumorigenesis, glutamine-derived 2-HG alters the epigenetic landscape of chromosomes and induces oncogenic transformation. Further investigations to explain the mechanism underlying glutaminolysis-induced metabolic reprogramming are needed. These efforts are anticipated to reveal new metabolic vulnerabilities of cancer cells that can be targeted by therapeutic interventions.
